# A simple method for preventing graft detachment during phacoemulsification cataract surgery after Descemet stripping automated endothelial keratoplasty: A case report

**DOI:** 10.1097/MD.0000000000013992

**Published:** 2019-01-11

**Authors:** Shiuh-liang Hsu, Po-yen Lee, Li-yi Chiu

**Affiliations:** Department of Ophthalmology, Kaohsiung Medical University Hospital, Kaohsiung Medical University, Kaohsiung, Taiwan.

**Keywords:** cataract surgery, Descemet stripping automated endothelial keratoplasty, phacoemulsification

## Abstract

**Rationale:**

Graft detachment and endothelial cell damages are 2 major concerns for cataract surgeries in cases after Descemet stripping automated endothelial keratoplasty (DSAEK). We invented a simple but innovative method to anchor the DSAEK graft with sutures during the surgery to avoid possible graft detachment.

**Patient concerns:**

We present a 59-year-old male who had done DSAEK surgery. Due to progressed blurry vision due to cataract, he underwent sequential cataract surgery 1 year after DSAEK surgery. We applied the method to the patient successfully and the result was satisfying.

**Diagnosis:**

Cataract post-DSAEK surgery.

**Interventions:**

At the beginning of surgery, we made 4 radial fixation sutures with Nylon 10-0 though cornea-DSAEK graft at paracentral point and then through limbus. The sutures were located at 45°, 135°, 225°, and 315°. At the end of surgery, 4 fixation sutures were removed.

**Outcomes:**

The DSAEK graft was kept well attached during and after the surgery. Only low-grade corneal edema was found postoperation.

**Lessons:**

We provide this special method and applied it to our patient successfully. By using our method, surgeons can reassure graft adhesion during and after surgery, especially for those need cataract surgery after DSAEK surgery.

## 1 Introduction

Because cataract surgery after endothelial keratoplasty is a challenging procedure, most surgeons decide to perform combined surgery or sequential phacoemulsification followed by Descemet stripping automated endothelial keratoplasty (DSAEK) in months.^[1,2]^ The possible risk for cataract surgery after DSAEK includes endothelial cell damage, graft detachment, or subsequent graft rejection.^[3]^ We use PubMed as database and search key words like phacoemulsification and DSAEK. Only 1 case report had been published discussing phacoemulsification after DSAEK.^[1]^ Also only 1 case series had been reported that phacoemulsification surgeries after DMEK were safe and can be performed with minimal risk for graft detachment.^[4]^ Here we present a simple but effective method to prevent graft detachment or dislocation during phacoemulsification surgery.

## 2 Case report

A 59-year-old patient had corneal decompensation after multiple episodes of herpetic endotheliitis. Due to minimal stromal involvement, DSAEK surgery was indicated and performed successfully. The vision improved from 0.02 to 0.1. However, cataract density increased soon in the surgical eye and vision decreased significantly (Fig. 2A). Therefore, cataract surgery was indicated and we decided to perform phacoemulsification and intraocular lens implantation for him.

**Figure 1:**
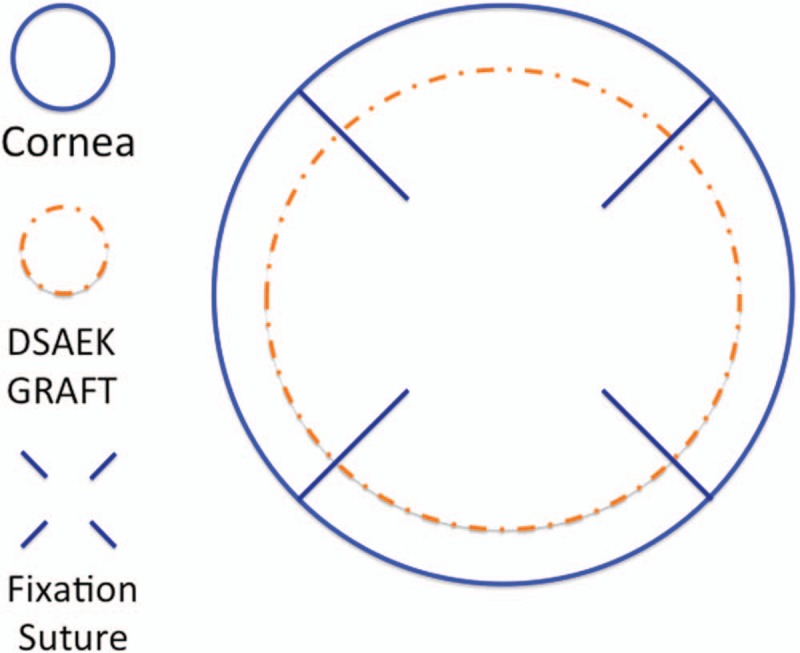
Four fixation radial sutures with Nylon 10.0 were applied in 4 directions: 45°, 135°, 225°, and 315°. The sutures were full layer including the graft and original host cornea originated from paracentral to limbus area. DSAEK = Descemet stripping automated endothelial keratoplasty.

At the beginning of cataract surgery, 4 fixation radial sutures with Nylon 10.0 were applied in 4 directions: 45°, 135°, 225°, and 315° (Fig. 1). The sutures were full layer including the graft and original host cornea originated from paracentral to limbus area (Fig. 1). This was aimed to prevent graft detachment or dislocation during the operation. After the fixation procedure, normal phacoemulsification lens extraction procedure was started. The main wound was designed at temporal side with clear cornea incision. About fluid dynamic, we adjusted the irrigation pressure to lower level. The irrigation function was kept off until the whole handpiece was well placed in the anterior chamber to avoid graft dislodgement. After lens extraction, a foldable intraocular lens was implanted. The 4 fixation sutures were removed at the end of the surgery.

**Figure 2:**
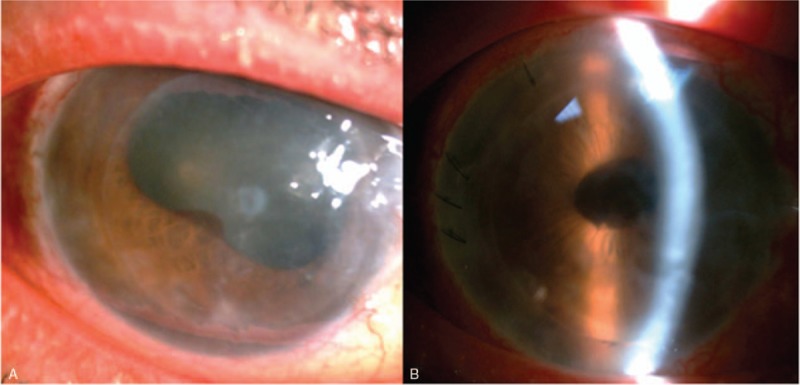
Cornea condition before (A) and after (B) cataract surgery. The graft was well attached during and after the operation. Postoperation day 1, only mild cornea edema was observed (B).

The operation was performed smoothly and the fixation sutures were removed at the end of the surgery. The graft was well attached during and after the operation. After the operation, only mild cornea edema was observed (Fig. 2B).

## 3 Discussion

There are 2 major concerns for cataract surgery after DSAEK or Descemet membrane autoamted endothelial keratoplasty (DMAEK): graft dislocation and endothelial cell damage. For DSAEK, some surgeons perform phacoemulsification before the surgery for steepening the anterior chamber and provide better surgical route for sequential DSAEK. Other majority performed combined lens extraction and DSAEK at the same time. However, for patients with clear lens who needed DSAEK of DMEK surgery, the incidence rate of cataract formation in 1 year after endothelial keratoplasty was high (40%).^[5]^ The incidence increased especially in cases of anterior chamber depth below 2.80 mm. Cataract surgeries may still be needed in the expecting expected future.

Even though no clinical cases had been reported having graft dislocation or detachment after sequential DSAEK and cataract surgery, the entire procedure still needs special caution to prevent graft dislocation and endothelial damage.

Only 1 case report had described detailed phacoemulsification procedure for post-DSAEK cataract, and the result was good.^[1]^

For better refraction outcome after the surgery, cataract surgeries after DSAEK provide more predictable refraction outcomes rather than combined surgeries. By using our method, surgeons can reassure graft adhesion during and after the surgery. There is no special learning curve for the surgeon. Our method is a safe choice especially for patients who needed cataract surgery soon after DSAEK.

There are some limitations to our study. This report consists of only 1 patient study and the follow-up duration was only 2 years. We may need further large size trial to approve the reproducibility of our method.

## Author contributions

**Conceptualization:** Shiuh-liang Hsu.

**Data curation:** Po-yen Lee, Li-yi Chiu.

**Formal analysis:** Shiuh-liang Hsu.

**Investigation:** Li-yi Chiu.

**Methodology:** Shiuh-liang Hsu.

**Resources:** Po-yen Lee.

**Supervision:** Shiuh-liang Hsu.

**Writing – original draft:** Li-yi Chiu.

**Writing – review & editing:** Po-yen Lee, Li-yi Chiu.

Li-yi Chiu orcid: 0000-0003-3915-7083.
